# Sap flow characteristics and responses to summer rainfall for *Pinus tabulaeformis* and *Hippophae rhamnoides* in the Loess hilly region of China

**DOI:** 10.1002/ece3.3639

**Published:** 2017-12-03

**Authors:** Xu Wu, Yakun Tang, Yunming Chen, Jie Wen, Yuli Xie, Senbao Lu

**Affiliations:** ^1^ State Key Laboratory of Soil Erosion and Dry‐Land Farming on the Loess Plateau Institute of Soil and Water Conservation Chinese Academy of Sciences Ministry of Water Resources Yangling Shaanxi China; ^2^ University of Chinese Academy of Science Beijing China; ^3^ Institute of Soil and Water Conservation Northwest Agriculture and Forestry University Yangling Shaanxi China; ^4^ College of Forestry Northwest Agriculture and Forestry University Yangling Shaanxi China

**Keywords:** anisohydric, *Hippophae rhamnoides*, isohydric, *Pinus tabulaeformis*, sap flow

## Abstract

As a major driving element of the structure and function of arid and semiarid ecosystems, rainfall is the essential factor limiting plant biological processes. To clarify the characteristics of transpiration and responses to summer rainfall, sap flow density (*F*_d_) of *Pinus tabulaeformis* and *Hippophae rhamnoides* was monitored using thermal dissipation probes. In addition, midday leaf water potential (ψ_m_) and leaf stomatal conductance (*G*_s_) were also analyzed to determine water use strategies. The results indicated that the diurnal variation in the normalized *F*_d_ values exhibited a single‐peak curve for *P. tabulaeformis*, while *H. rhamnoides* showed multiple peaks. The normalized *F*_d_ for *P. tabulaeformis* remained relatively stable regardless of rainfall events. However, there was also a significant increase in the normalized *F*_d_ for *H. rhamnoides* in response to rainfall in June and August (*p *< .05), although no significant differences were observed in July. The normalized *F*_d_ values for *P. tabulaeformis* and *H. rhamnoides* fitted well with the derived variable of transpiration, an integrated index calculated from the vapor pressure deficit and solar radiation (*R*_s_), using an exponential saturation function. The differences in fitting coefficients suggested that *H. rhamnoides* showed more sensitivity to summer rainfall (*p *< .01) than *P. tabulaeformis*. Furthermore, during the study period, *P. tabulaeformis* reduced *G*_s_ as soil water decreased, maintaining a relatively constant ψ_m_; while *H. rhamnoides* allowed large fluctuations in ψ_m_ to maintain *G*_s_. Therefore, *P. tabulaeformis* and *H. rhamnoides* should be considered isohydric and anisohydric species, respectively. And more consideration should be taken for *H. rhamnoides* in the afforestation activities and the local plantation management under the context of the frequently seasonal drought in the loess hilly region.

## 1 INTRODUCTION

As a major driving element of the structure and function of arid and semiarid ecosystems, rainfall is the essential factor limiting plant biological processes (Cao, Jiang, Zhang, Zhang, & Han, 2011; Moran et al., 2009). Furthermore, global climate change appears to increase the variability of rainfall patterns in these regions. As a result, plants may endure recurring cycles of water scarcity followed by rainfall events (Jackson et al., 2001; Smith & Nowak, 1990). Understanding the mechanisms that underlie plant responses to rainfall is a key to understand how global climate change will affect arid and semiarid ecosystems (Jiang, 2001; Yuan & Deng, 2004).

Sap flow is a direct indicator of tree transpiration, and it can reflect the physiological characteristics and water use response of individual trees to environmental factors (Wang & Wang, 2012). Some studies have shown that sap flow significantly accelerates after trees absorb water provided by rainfall events (Schwinning & Sala, 2004), but this influence may be related to multiple factors, such as tree species (Cheng et al., 2006) and rainfall amount (Ivans, Hipps, Leffler, & Ivans, 2006). For example, shallow‐rooted *Isopogon gardneri* rapidly increased transpiration up to fivefold after a 34 mm rain event in southern Australia, whereas deep‐rooted *Eucalyptus* species were sufficiently reliant on antecedent soil water and did not respond to summer rainfall (Burgess, 2006). Zeppel, Macinnis‐Ng, Ford, and Eamus (2008) found that rainfall pulses of <20 mm did not significantly increase water use of *Eucalyptus callitris*. In contrast, a rainfall threshold of 10 mm induced a significant response in mesquite shrubs (Fravolini et al., 2005). Pataki, Oren, and Smith (2000) indicated that broad‐leaf species, such as *Populus tremuloides*, showed the greatest increases in sap flow density (*F*_d_) with increasing atmospheric water demand, while the coniferous species *Pinus contorta* showed the lowest *F*_d_. The stomatal regulation of transpiration and the hydraulic structure of plants may account for their different behavior in response to changes in water conditions (Franks, Drake, & Froend, 2007; Hölscher, Koch, Korn, & Leuschner, 2005).

Plants fall into two categories across the continuum of stomatal regulation of water use: isohydric and anisohydric (Tardieu & Simonneau, 1998). Isohydric species, such as *Pinus edulis*, reduce stomatal conductance (*G*_s_) as soil water decreases, maintaining relatively constant leaf water potential (ψ_m_) (West, Hultine, Sperry, Bush, & Ehleringer, 2008; Williams & Ehleringer, 2000). Anisohydric species, such as *Juniperus monosperma*, allow large fluctuations in ψ_m_, sustaining higher *G*_s_ than isohydric species (Tardieu & Simonneau, 1998; West et al., 2008). Compared with isohydric species, anisohydric species tend to occupy more drought‐prone habitats to some extent, but under particularly intense droughts or prolonged drought duration, anisohydric species may experience xylem embolism or even mortality (McDowell et al., 2008). Therefore, understanding the characteristics of water use and the responses of tree species to rainfall in arid and semiarid regions will help to build reasonable vegetation restoration models as rainfall patterns change.

The Loess Plateau, located in upper‐middle reaches of the Yellow River in northern China, has a number of serious soil erosion challenges, which are largely caused by intensive and unsustainable human activities (Lu & van Ittersum, 2004). Previous studies have indicated that plantations play important roles in reducing soil loss and water loss and maintaining ecological functions (Xu, Xu, Huang, Shan, & Li, 2011). *Pinus tabulaeformis* and *Hippophae rhamnoides* are the dominant woody species on the Loess Plateau, and both species have been widely used for ecological restoration (Chen, Liu, & Hou, 2002; Wu & Yang, 1998). In recent years, however, tree species have experienced water shortages during the course of their life cycle because of low rainfall and high atmospheric evaporative demands in the region (Wang, Fu, Gao, Liu, & Zhou, 2013). Some recent studies have focused on sap flow characteristics and its influencing factors of *P. tabulaeformis* and *H. rhamnoides* (Wu, 2016; Zhang, Wei, & Chen, 2015), but little is currently known about the water use strategies and responses to rainfall for these two species. Analyzing the different water use responses of plantation species in the Loess hilly region to summer rainfall can provide a scientific basis for local plantation management in terms of tree water use during ecological restoration (Blackman & Brodribb, 2011).

In this study, we investigated the response to rainfall and the water use types of *P. tabulaeformis* and *H. rhamnoides*, by measuring *F*_d_, ψ_m_, and *G*_s_ under field conditions. Our aims were to (1) understand the different sap flow responses of these two tree species to summer rainfall and (2) analyze their patterns of stomatal regulation to identify their water use types.

## 2 MATERIALS AND METHODS

### 2.1 Study site

The experiments were conducted at the Ansai Station of the Chinese Academy of Sciences, located in Shaanxi Province, China (36°51′N, 109°19′E, elevation ranges from 1068 to 1309 m a.s.l.). The climate is a typical temperate conditional monsoon; mean annual rainfall amounts are about 500 mm, with great seasonal variations (Shan & Chen, 1993). Average annual temperature is 8.8°C, with a low of −6.9°C in January and a high of 22.6°C in July. The range of average annual potential evaporation is 600–800 mm. The soil type is classified as Calcic Cambisols that developed on wind‐deposited loess parent material (Wang, Fu, Qiu, & Chen, 2003). The main woody plants are *P. tabulaeformis*,* Robinia pseudoacacia*,* H. rhamnoides*, and *Caragana korshinskii*, and the main herb species are *Artemisia gmelinii* and *Stipa bungeana*.

We selected two adjacent stands for the experiment in pure plantations of *P. tabulaeformis* and *H. rhamnoides* at an elevation of 1268 m a.s.l. General characteristics of the two study plots and individual trees in this study are presented in Table 1.

**Table 1:** Characteristics of the sample trees

Species	*P. tabulaeformis*	*H. rhamnoides*
Height (m)	3.39 ± 0.09	3.30 ± 0.19
Diameter at breast/ground height (DBH/DGH, cm)	6.59 ± 0.54	4.92 ± 0.44
Sapwood thickness (cm)	2.24 ± 0.20	0.95 ± 0.34

### 2.2 Sap flow density measurements

#### 2.2.1 Probe installation

Sap flow densities were measured in seven *P. tabulaeformis* and six *H. rhamnoides* individuals in two stands using thermal dissipation probes from June to August 2015. The probe used for *P. tabulaeformis* was 2 mm in diameter and 30 mm in length; the probe used for *H. rhamnoides* was 2 mm in diameter and 10 mm in length. After peeling off two pieces of bark (20 mm × 20 mm), the probes were inserted into sapwood about 0.15 m apart vertically at DBH (1.3 m) of *P. tabulaeformis* and DGH (0.3 m) of *H. rhamnoides*. The upper probe included a heater that was supplied with a constant power of 0.15 W, and the lower probe was unheated for reference (James, Clearwater, Meinzer, & Goldstein, 2002). The temperature difference between two probes was measured every 30 s, and 30 min averages were recorded on a data logger (CR1000; Campbell Scientific Inc., Logan, UT, USA) with a multiplexer (AM16/32A; Campbell Scientific). The sensor was mounted with waterproof silicone and covered with an aluminum box cover to avoid physical damage and thermal influences from radiation (Wu, Chen, & Tang, 2015).

According to the empirical relationship between sap flow and the temperature difference between the probes established by Grainer and revalidated by other studies (Clearwater, Meinzer, Andrade, Goldstein, & Holbrook, 1999; Granier, 1987), the uncorrected sap *F*_d_ was calculated as:

(1) $$F_{d}=119\times {\left[\frac{\Delta T_{\max}-\Delta T}{\Delta T}\right]}^{1.231}$$

where *F*_d_ is sap flow density (ml m^−2^ s^−1^), ∆*T* is the temperature difference between the two probes, and ∆*T*_max_ is the maximum value of ∆*T* recorded at the no‐transpiration state when *F*_d_ is zero.

#### 2.2.2 Calibration of the original‐type Granier sensors

There has been concern about the validity of the empirical formula when the sensor design deviates from the original‐type Granier sensors (Lu, Urban, & Zhao, 2004). In practice, there are three situations where differences in the sapwood (*D*_sw_) and the length of the probe may lead to a deviation from the true *F*_d_ value:

The ideal case is when the *D*_sw_ depth is equal to the length of the probe, which means that *F*_d_ can be calculated using Formula (1).

If the length of the probe is less than *D*_sw_, the ideal case calculation can be used if *F*_d_ is assumed to be the same in the sapwood beyond the length of the probe. This assumption may not always be true as there is often a substantial variation in *F*_d_ across the whole cross section.

If the length of the probe is greater than the *D*_sw_, the probe does not provide a true *F*_d_ value, and the measured *F*_d_ always underestimates the true *F*_d_ value. Based on a similar analysis, Clearwater et al. (1999) proposed an alternative method to calculate *F*_d_ in the active sapwood. If parts of the probe are inserted into nonconducting xylem while the remainder is in sapwood with a relatively uniform *F*_d_, then it can be assumed that the measured ∆*T* is a weighted mean for ∆*T* in the sapwood (∆*T*_sw_) and ∆*T* in the inactive xylem (∆*T*_max_):

(2) $$\Delta T=a\Delta T_{\mathrm{sw}}+b\Delta T_{\max}$$

where *a* and *b* are the parts of the probe in the sapwood and inactive xylem (*b *=* *1–*a*), respectively. This approach assumes that the thermal properties of inactive xylem are the same as sapwood when *F*_d_ = 0. If the depth of the sapwood is known, then the corrected *F*_d_ for that portion of the sapwood can be calculated by replacing ∆*T* in Formula (2) with ∆*T*_sw_.

There has also been a recommendation that species‐specific calibration should be applied to the Granier‐type probes data (Lu et al., 2004; Smith & Allen, 1996). Furthermore, a recent report suggests that *F*_d_ is significantly underestimated by heat techniques, particularly by the thermal dissipation method (Du et al., 2011; Steppe, De Pauw, Doody, & Teskey, 2010). In this study, we calculated *F*_d_ by correcting the original Granier data and normalizing the data to improve the validity of the empirical formula when the sensor design deviated from the original. Furthermore, the differences among replicated trees within species were minimized. The normalized *F*_d_ was determined by dividing all sap flow data for each replicate tree by the maximum recorded over the 3 months. Consequently, each replicate individual has a maximum normalized *F*_d_ of 1.0, and averages can be reasonably calculated for replicates within species. Therefore, all normalized *F*_d_ data in this paper represent the mean values of each sample tree for *P. tabulaeformis* and *H. rhamnoides*, respectively.

### 2.3 Environmental factors measurements

Meteorological data were obtained from the meteorological station located close to the study site (ca. 200 m). Solar radiation (*R*_s_) was measured by a quantum sensor (LI‐190SZ; Li‐COR, Lincoln, NE, USA), air temperature and relative humidity were measured by a thermo sensor (HMP45D; Vaisala, Helsinki, Finland), and rainfall amount was monitored using a rain gauge (DRD11A Rain Detector). In addition, soil volumetric moisture content was measured through EM50 (ECH_2_O; Decagon Devices, Inc. Pullman, WA, USA), installed at five depths (10, 20, 50, 100, and 150 cm) below the soil surface in the *P. tabulaeformis* and *H. rhamnoides* plots. These parameters were all stored as 30 min values.

### 2.4 Measurement of leaf water potential and stomatal conductance

The transpiration characteristics of the species were investigated by measuring the daytime temporal change patterns for canopy conductance and leaf stomatal conductance. Canopy conductance (*G*_t_) changes were estimated using the sap flow data as presented below. Leaf *G*_s_ was measured in situ for canopy sunlit leaves using a Li‐6400XT portable photosynthesis system (LI‐COR) under approximately natural conditions. The ψ_m_ was measured at 11:00–13:00 using a Pressure Chamber (1515D; PMS Instruments, and Corvallis, OR, USA) for both species. In each measurement, three fully extended healthy leaves were selected from three replicate trees.

### 2.5 Investigation of the root systems

Excavation and drilling methods were used to measure the root systems of the two species in order to investigate their functional types and water use strategies. The roots were sampled from 0 to 2 m in depth at 0.1 m intervals.

### 2.6 Data analyses

To investigate the response of sap flow to rainfall in *P. tabulaeformis* and *H. rhamnoides* from June to August, three rainfall events were selected, and the sap flow characteristics were compared before and after rainfall to analyze changes in water use for the two species. We chose a derived variable of transpiration (VT) to represent *R*_s_ and VPD, which was calculated as a simplified combination of *R*_s_ and VPD (Iida, Nakatani, & Tanaka, 2006).

(3) $$\text{VT}=\text{VPD}\times R_{s}^{1/2}$$

where VPD is the vapor pressure deficit (kPa), and *R*_s_ is solar radiation (W/m^2^).

Previous researchers have shown that the relationship between *F*_d_ and VT can reflect the response patterns of sap flow to atmospheric factors for each species under different soil water conditions after rainfall events (Du et al., 2011). They analyzed the relationship between *F*_d_ and VT using the following exponential saturation function (Ewers, Mackay, & Samanta, 2007; Kumagai, Tateishi, Shimizu, & Otsuki, 2008):

(4) $$F_{d}=a\left(1-\text{exp}\left(-b\text{VT}\right)\right)$$

where VT is the variable of transpiration, *F*_d_ is the normalized sap flow density, and *a* and *b* are the fitting parameters. The differences in fitting coefficients were used to roughly divide the species into two types based on their rainfall sensitivity (Du et al., 2011). According to Oren, Zimmermann, and Terbough (1996), the hydraulic conductance from soil to atmosphere can be estimated from the slope of the *F*_d_–VT relationship. A steep slope in the relationship indicates a high conductance. This is consistent with the model coefficient (parameter *b*) (Du et al., 2011; Ewers et al., 2007).

The normalized *F*_d_ was also used to estimate the relative canopy conductance. The total canopy conductance of individual trees is usually estimated from the ratio between the whole‐tree *F*_d_ and VPD. The calculation also includes a unit conservation coefficient (Kostner et al., 1992):

(5) $$G_{t}=k\times E/\text{VPD}$$

where *G*_*t*_ is total canopy conductance (μm/s), *E* is the whole‐tree *F*_d_ (μm/s), *k* is the unit conversion coefficient (kPa) calculated from the water density, gas constant for water vapor, and air temperature. As we used a normalized *F*_d_ in this study, we calculated a simplified relative canopy conductance as follows:

(6) $$G_{t}=F_{d}/\text{VPD}$$

In addition, this is because VPD usually contributes more than two‐thirds of the total transpiration, making it the dominant environmental variable, with the remainder from the radiation component (Green, 1993; Zhang, Simmonds, Morison, & Payne, 1997). Therefore, combined formulas of (3) and (6), the law of stomatal regulation can be roughly determined by the following formula:

(7) $$G_{t}=F_{d}/\text{VPD}\approx F_{d}/\left(\text{VT}/R_{s}^{1/2}\right)\approx F_{d}/\text{VT}$$

One‐way analyses of variance (ANOVA) was used to compare significant differences in the responses of sap flow to rainfall events for these two tree species and reanalyze the significant differences in leaf water potential (ψ_m_) and stomatal conductance (*G*_s_) within species. In addition, the general linear model was used to test the significance of the fitting curve between *F*_d_ and VT before and after the rainfall events. All statistical analyses were performed using SPSS 16.0 (SPSS Inc., Chicago, IL, USA); *p < *.05 was considered statistically significant. All figures were plotted using SigmaPlot 10.0 (Systat Software Inc., San Jose, CA, USA).

## 3 RESULTS

### 3.1 Environmental factors, root distribution, and sap flow density characteristics

**Figure 1:**
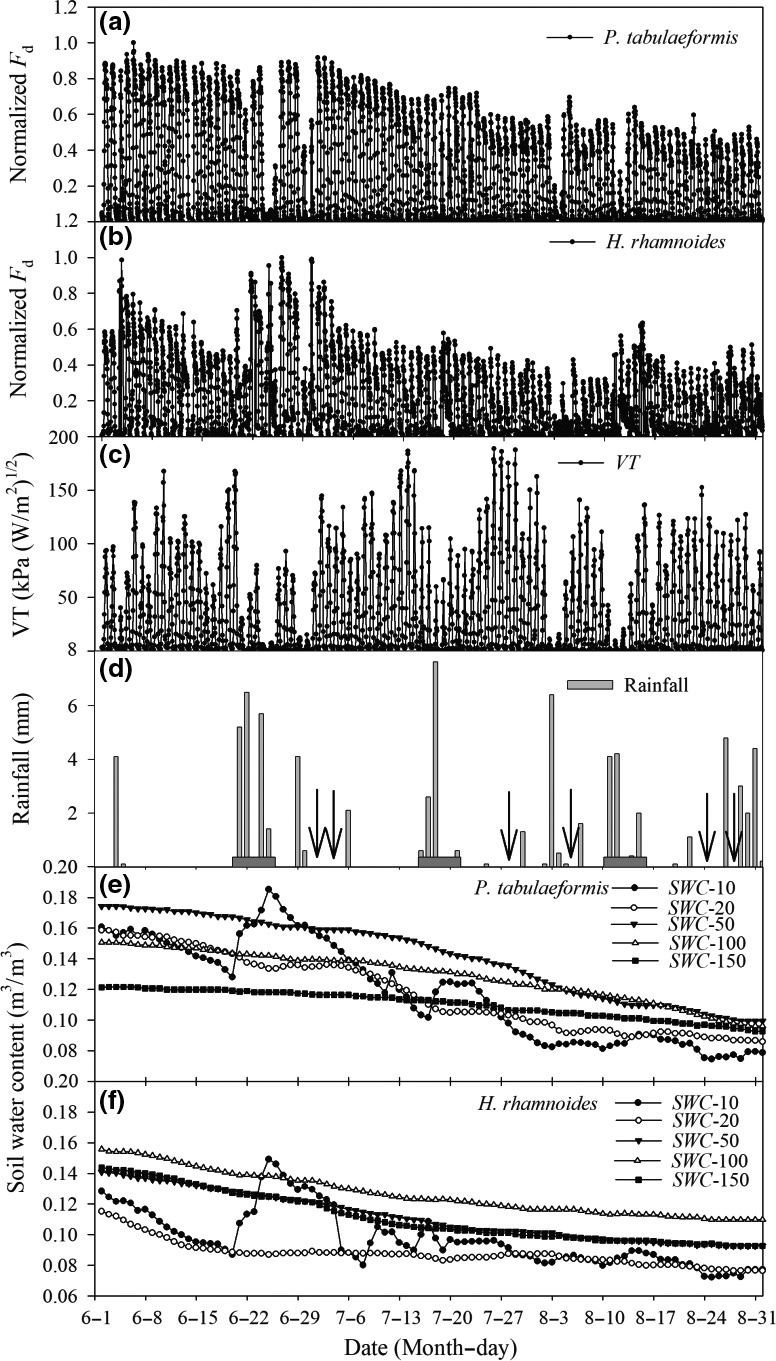
Daily values for (a) and (b), the normalized sap flow density (*F*_d_) during whole experiment period (1 June to 31 August), data represent the mean values of each sample tree for *Pinus tabulaeformis* and *Hippophae rhamnoides*, respectively. (c), daily values of the derived variable of transpiration (VT). (d), daily rainfall, and the three rectangular boxes on the *x* axis are the research time periods, with the center of selected rainfall events on 20 to 24 June (18.8 mm), 15 to 20 July (11.6 mm), and 10 to 14 August (10.9 mm). Arrows represent the observed time of the leaf water potential and stomatal conductance. (e) and (f), *SWC*10, *SWC*20, *SWC*50, *SWC*100, and *SWC*150 are soil volumetric moisture content (m^3^/m^3^) at 10, 20, 50, 100, and 150 cm below the soil surface for *P. tabulaeformis* and *H. rhamnoides* plot, respectively

The diurnal courses of the normalized *F*_d_ and VT values for these two tree species from June to August are shown in Figure 1a–c. In most cases, high normalized *F*_d_ values coincided with high VT values (Figures 1a–c). For example, on 5 June, normalized *F*_d_ values were 0.431 and 0.326 for *P. tabulaeformis* and *H. rhamnoides*, respectively. In general, on days when rain occurred resulted in low VT, normalized *F*_d_ values were greatly reduced. For instance, on 4 August, a sudden rainfall event occurred, which led to low VT values, and the normalized *F*_d_ values were 0.049 and 0.056, respectively. The daily patterns of transpiration for these two tree species showed relatively lower values in late July and August, although the VT values were high (Figure 1c).

**Figure 2:**
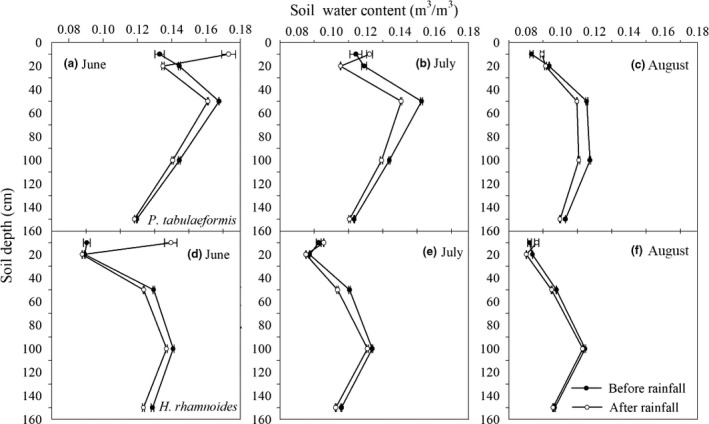
Soil volumetric water content along vertical profiles (a–c) in *Pinus tabulaeformis* and (d–f) *Hippophae rhamnoides* plot, respectively, collected three days during before and after rainfall events (mean ± *SE*) over the experimental periods, respectively

The rainfall and soil moisture conditions from June to August are shown in Figure 1d–f. During the study period, there were 33 rainfall events, which produced a total rainfall of 78 mm and accounted for 35% of the annual rainfall. Rainfall had a greater effect on the soil moisture content at 10 cm depth more frequently than on the other four soil depths in the two study plots. Soil water content below 20 cm greatly decreased between June and August, which might not be significantly recharged by the rainfall events. Soil water content at 10 cm depth responded to rainfall events if the cumulative rainfall total over a 3‐ to 5‐day period exceeded 10 mm. A single rainfall event of <10 mm had little effect on soil water conditions (Figure 1d–f). Data collected along vertical profiles in the two study plots both before and after rainfall showed that rainfall had a significant effect on surface soil moisture, and the short‐term changes in soil moisture below a depth of 50 cm were subject to little influence from rainfall during the study period (Figure 2).

**Figure 3:**
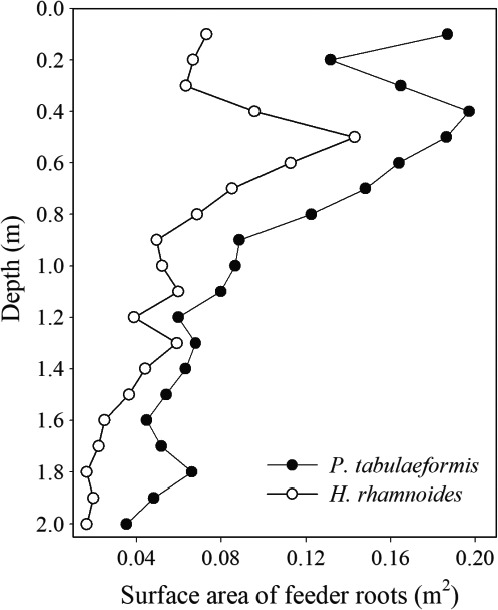
Vertical distribution of fine roots of *Pinus tabulaeformis* and *Hippophae rhamnoides*

The vertical distribution of fine roots in the root system is shown for the two tree species and is based on the surface area of the lateral roots in each 10 cm soil section (Figure 3). The root distribution with soil depth showed that *P. tabulaeformis* roots tended to be concentrated at around 10 and 40 cm depth, *H. rhamnoides* roots were concentrated around 50 cm depth. Furthermore, the surface area of the *P. tabulaeformis* fine roots was greater than *H. rhamnoides* at the same soil depth.

### 3.2 Sap flow density and its relationship with VT after rainfall events

#### 3.2.1 The response of sap flow density to rainfall

**Figure 4:**
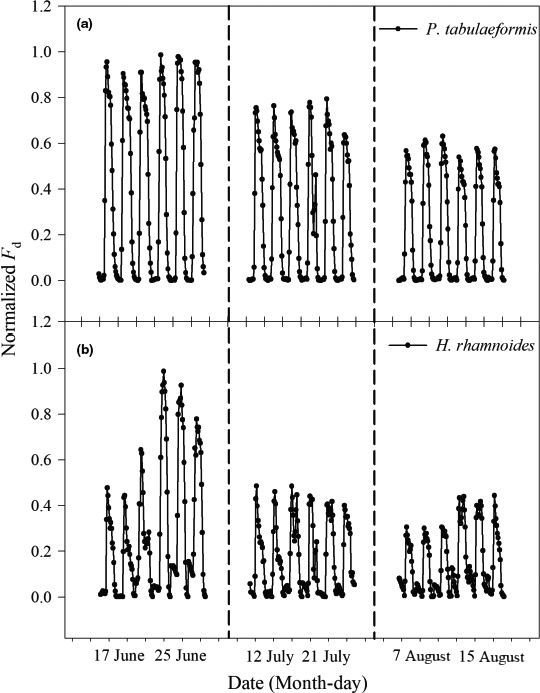
Changes in normalized *F*_d_ of (a) *Pinus tabulaeformis* and (b) *Hippophae rhamnoides* during each before and after rainfall period over the study period. The different periods are separated by dotted lines

The diurnal courses of the normalized *F*_d_ values exhibited a single‐peak curve for *P. tabulaeformis*, while *H. rhamnoides* exhibited a multipeak curve before and after rainfall (Figures 4a,b). The normalized *F*_d_ values were approximately zero between 0:00 and 6:00 hr, and it increased shortly after sunrise. The normalized *F*_d_ values decreased to a relatively lower level after sunset and gradually reached the minimum after midnight. The diurnal courses of the normalized *F*_d_ for *P. tabulaeformis* remained relatively stable before and after rainfall events in the three periods (Figure 4a). The normalized *F*_d_ for *H. rhamnoides* after rainfall was significantly higher than that before rainfall (except in July) (Figure 4b).

**Figure 5:**
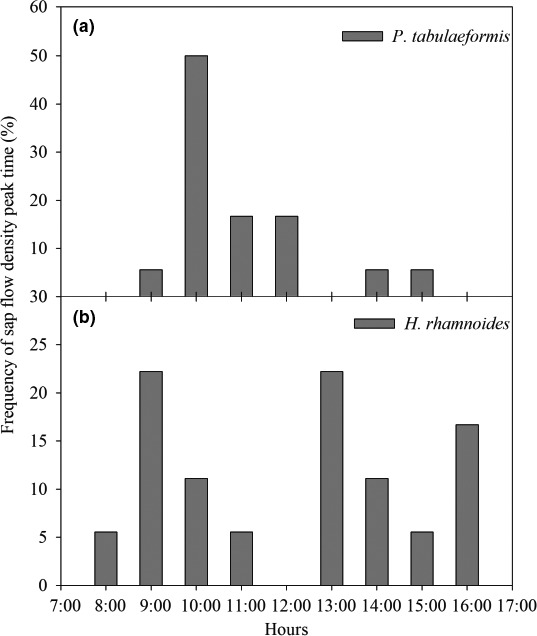
Frequency of normalized *F*_d_ peak times of (a) *Pinus tabulaeformis* and (b) *Hippophae rhamnoides* during the research periods

In order to further explore the differences in the pattern of daytime normalized *F*_d_ values between these two tree species, we counted the frequency of *F*_d_ peak times during the study periods (Figure 5). The results showed that the *F*_d_ peak time of *P. tabulaeformis* was mainly concentrated at around 10:00 (9:30–10:30) (Figure 5a). *H. rhamnoides* had a relatively wide band of *F*_d_ peak time distribution, a little more than 1/5 of peaks appeared around 9:00 and 13:00, and about 1/5 of peaks appeared around 15:30 (15:00–16:00) (Figure 5b).

**Figure 6:**
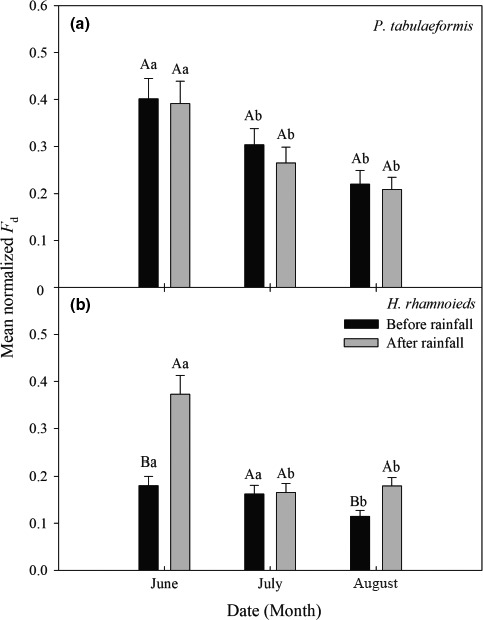
Normalized *F*_d_ of (a) *Pinus tabulaeformis* and (b) *Hippophae rhamnoides* during each before and after rainfall period over the 3 months (mean ± *SE*). Capital letters indicate the significant difference between before and after rainfall at 0.05 levels, and small letters indicate the significant difference among months for before and after rainfall events at 0.05 levels

There was no obvious increase in the normalized *F*_d_ for *P. tabulaeformis* in response to the three rainfall events. The normalized *F*_d_ for *P. tabulaeformis* reached its highest level in June before and after rainfall periods, and there was a significant difference between July and August, but no significant difference between July and August (Figure 6a). There was a significant increase in the normalized *F*_d_ for *H. rhamnoides* after the rainfall in June and August (except in July). The normalized *F*_d_ of *H. rhamnoides* also reached its highest level in June, and there was a significant difference between June and August, but there was no significant difference between June and July before rainfall events. However, after rainfall periods, there was a significant difference between June and other 2 months (July and August), but there was no significant difference between July and August (Figure 6b).

#### 3.2.2 Relationship between normalized *F*_d_ and VT in response to rainfall

**Figure 7:**
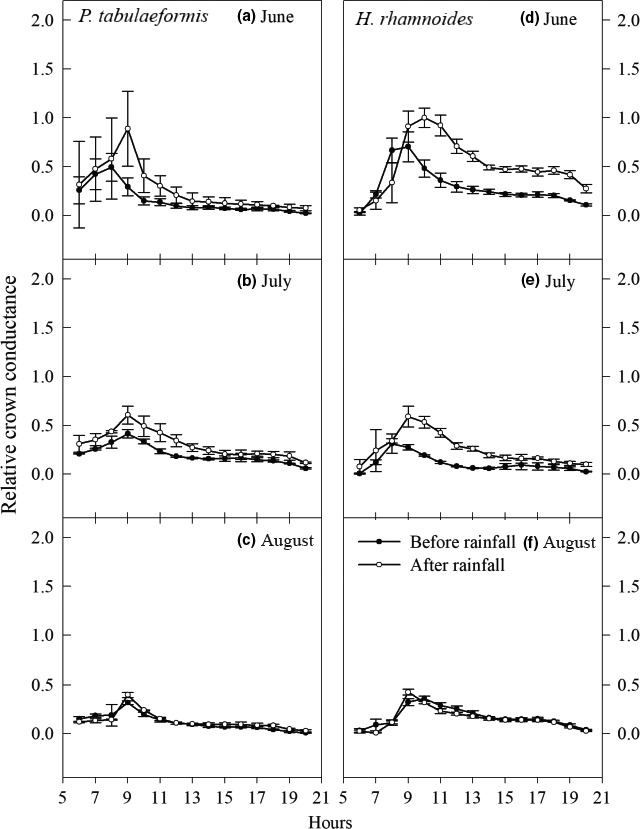
Relative crown conductance was estimated by dividing normalized *F*_d_ by *VPD* and was presented as mean ± *SE* for each sample tree of (a–c) *Pinus tabulaeformis* and (d–f) *Hippophae rhamnoides*, respectively

The diurnal courses of the estimated relative canopy conductance for *P. tabulaeformis* and *H. rhamnoides* had a single‐peak curve before and after rainfall (Figure 7). These two tree species showed rapid increases and decreases in canopy conductance during the morning, but remained low in the afternoon. However, *P. tabulaeformis* reached its maximum earlier than *H. rhamnoides,* and this was followed by a steep decline before and after rainfall. By midday, the canopy conductance for this species had fallen to levels that were lower than those recorded in the early morning (e.g., 6:00–7:00). *H. rhamnoides* showed gradual declines after they had peaked and maintained their conductance at or above the levels recorded during the early morning.

**Figure 8:**
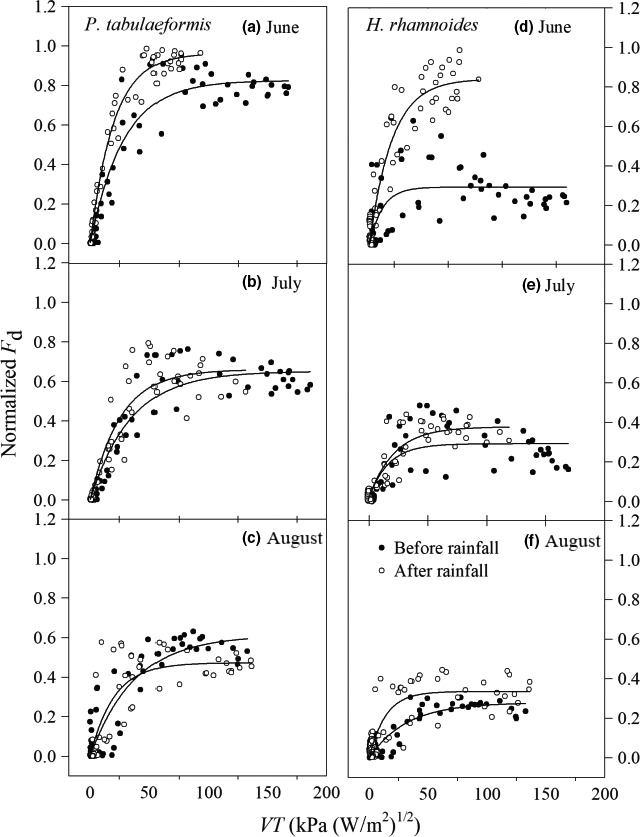
Relationship between normalized *F*_d_ and variable of transpiration (VT) of (a–c) *Pinus tabulaeformis* and (d–f) *Hippophae rhamnoides* during each before and after rainfall event for June, July, and August

To further elucidate the response patterns of *F*_d_ to atmospheric factors for these two tree species under different soil water conditions, data sets of normalized *F*_d_ and VT values were analyzed before and after rainfall over 3 months. The normalized *F*_d_ values for *P. tabulaeformis* and *H. rhamnoides* correlated with transpiration (VT) using an exponential saturation function (Formula (4)). Generally, *F*_d_ increased in response to rising VT, while these values tended to stabilize when VT reached about 50 kPa (W/m^2^)^1/2^ (Figure 8).

Table 2 summarizes the analysis reports for normalized *F*_d_ and VT regression fitting, the variations in model coefficients (*a* and *b*), and related significance tests. There was a relatively large difference in the variation coefficients (*a* and *b*) before and after rainfall periods for *H. rhamnoides*, which suggests the transpiration process is sensitive to soil water conditions (Table 2). In contrast, there were lower variation coefficient differences before and after rainfall, for *P. tabulaeformis,* suggesting less amplitude in the response of transpiration to the soil water changes (Figure 8). Parameter b was greater in *H. rhamnoides* than in *P. tabulaeformis* for each of the three rainfall events, suggesting a high hydraulic conductance for *H. rhamnoides* (Table 2).

**Table 2:** Regression analysis results for sap flow density and variation in transpiration (VT) for *P. tabulaeformis* and *H. rhamnoides* before and after rainfall events during June, July, and August

Species	Month	Before rainfall	After rainfall	The variations in coefficient *a* and *b* (%)	Difference between coefficients
*P. tabulaeformis*	June	*a *=* *49.073*b *=* *0.035*R* ^2^ = .9343*p *<* *.0001	*a *=* *56.490*b *=* *0.047*R* ^2^ = .9815*p *<* *.0001	15.1134.29	*p *<* *.001*p *<* *.01
	July	*a *=* *37.638*b *=* *0.029*R* ^2^ = .9115*p *<* *.0001	*a *=* *37.585*b *=* *0.039*R* ^2^ = .8960*p *<* *.0001	−0.1434.48	*p *<* *.05*p *<* *.05
	August	*a *=* *34.596*b *=* *0.028*R* ^2^ = .8568*p *<* *.0001	*a *=* *26.992*b *=* *0.049*R* ^2^ = .7859*p *<* *.0001	−21.9875	*p *<* *.001*p *<* *.01
*H. rhamnoides*	June	*a *=* *5.6346*b *=* *0.051*R* ^2^ = .6621*p *<* *.0001	*a *=* *11.3044*b *=* *0.092*R* ^2^ = .9270*p *<* *.0001	100.6280.39	*p *<* *.001*p *<* *.001
	July	*a *=* *5.521*b *=* *0.042*R* ^2^ = .8279*p *<* *.0001	*a *=* *6.415*b *=* *0.052*R* ^2^ = .9170*p *<* *.0001	16.1923.81	*p *<* *.05*p *<* *.05
	August	*a *=* *5.025*b *=* *0.041*R* ^2^ = .701*p *<* *.0001	*a *=* *6.128*b *=* *0.091*R* ^2^ = .761*p *<* *.0001	21.95121.95	*p *<* *.001*p *<* *.001

*C* = ((*B*−*A*)/*A*) × 100% (where *C* is the relative variations in coefficient *a* and *b*,* A* and *B* is the fitting coefficient of before and after rainfall periods, respectively.)

### 3.3 Stomatal regulation patterns

**Figure 9:**
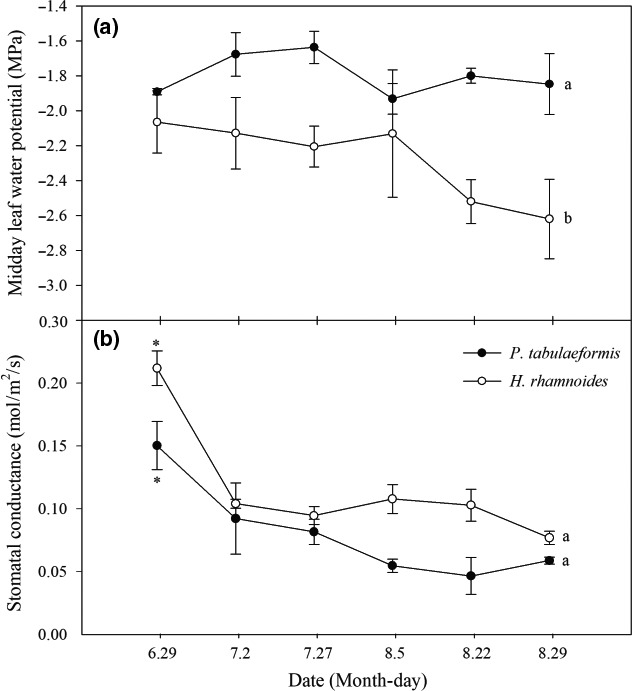
(a) Midday leaf water potential for *Pinus tabulaeformis* (filled symbols) and *Hippophae rhamnoides* (open symbols), and (b) stomatal conductance during the study period. Different lowercase letters indicate the significant differences among species at .05 levels. “*” indicates the significant differences among measurements over 3 months at .05 levels for specific species. The data represent mean ±*SE*

During the study period, there were no significant differences in ψ_m_ between the different measurement periods for *P. tabulaeformis* and *H. rhamnoides*. The ψ_m_ of *P. tabulaeformis* was approximately −1.7 MPa, and the coefficient of variation was 8.34%. The ψ_m_ of *H. rhamnoides* had a wide range of −2.06 to −2.62 MPa, and the coefficient of variation was 13.19%. The ψ_m_ in *P. tabulaeformis* was significantly higher than that in *H. rhamnoides* (*p *<* *.05) (Figure 9a). In contrast, there was a significant difference in *G*_s_ between June and the other 2 months, but there was no significant difference between the other 2 months. The average *G*_s_ of *H. rhamnoides* (0.12 mol/m^2^/s^1^) was 1.5 times greater than that in *P. tabulaeformis* (0.08 mol/m^2^/s^1^); however, the coefficient of variation of *H. rhamnoides* (16.25%) was significantly lower than *P. tabulaeformis* (45.51%) (Figure 9b). This indicated that as soil water changes (Figure 1e,f), *P. tabulaeformis* may reduce *G*_s_ to maintain a relatively constant ψ_m_, whereas *H. rhamnoides* may allow large fluctuations in ψ_m_ to maintain *G*_s_.

## 4 DISCUSSION

### 4.1 Sap flow characteristics

Typical daytime *F*_d_ patterns for tree species may indicate water use strategies (Du et al., 2011). Yin, Cheng, and Zhang (2011) showed that the daily changes of *F*_d_ for *P. tabulaeformis* were in the form of a single‐peak curve that reached a peak at 12:00–14:00. Our results also showed that *F*_d_ exhibited a single‐peak curve for *P. tabulaeformis* (Figure 4a), and the peak was mainly concentrated at 10:00 (Figure 5a). The diurnal variation of *F*_d_ for *H. rhamnoides* exhibited a multipeak curve (Figure 4b), with peaks concentrated at 9:00, 13:00, and 16:00, which implies active sap flow in this species until late afternoon (Figure 5b). Yu, Yang, Zang, and Xu (2008) found similar results for *H. rhamnoides* in Huangfuchuan Basin, but Shen et al. (2014) showed a typical single‐peak pattern for *H. rhamnoides*. This may depend on meteorological factors and water conditions. Furthermore, environmental factors affect broad‐leaf species more than coniferous species (Cheng et al., 2015; Yang et al., 2012). The diurnal courses of the normalized *F*_d_ for *P. tabulaeformis* remained relatively stable before and after rainfall events over the 3 months (Figure 4a). The normalized *F*_d_ for *H. rhamnoides* (Figure 4b) after rainfall was significantly higher than before rainfall (except in July), which indicated significant differences in the responses of these two tree species to the same rainfall event. This might be related to the anatomical structure of the xylem in these two tree species. Zhai, Li, and Nie (2003) indicate that the *P. tabulaeformis* belongs to nonpore wood and the xylem conducting tissues that are composed of tracheids, whereas the *H. rhamnoides* belongs to porous wood and has an advanced form of xylem conducting tissue that is composed of vessel elements (Ai, Li, Chen, & Chen, 2015). According to the Hagen–Poiseuille law, the value for sap flow is proportional to the fourth power of the hydraulic radius (Tyree & Zimmermann, 2002). Therefore, in the water transport process, the resistance in *P. tabulaeformis* was relatively greater than in *H. rhamnoides*. Furthermore, the canopy structure and the hydraulic architecture of roots may also be the reason for the sensitive response differences to rainfall events among tree species (Cheng et al., 2015; Zhu et al., 2010). Extensive investigations of the hydraulic architecture characteristics of *P. tabulaeformis* and *H. rhamnoides* are needed to clarify the difference in water use by these two tree species.

### 4.2 Sap flow responses to rainfall

The function of exponential saturation has been used to elucidate the response types of *F*_d_ to atmospheric water demand for tree species (Ewers et al., 2007; Kumagai et al., 2008). In a comparative study on three forest species in the semiarid Loess Plateau region of China, large differences were found in the fitting parameters during before and after rainfall periods in *R. pseudoacacia*, which suggests that the transpiration process is sensitive to soil water conditions. In contrast, the other species showed less amplitude in the response of transpiration to soil drought (Du et al., 2011). Further study on the effects of stomatal conductance on tree water use will improve understanding of the eco‐hydrological relationships across large spatial scales because stomatal conductance responds to various environmental factors, such as solar radiation, vapor pressure deficit, and the water status of the root (Buckley, 2005). In general, stomatal conductance increased as the atmospheric evaporative demands rose, but when the atmospheric evaporative demands increased continuously up to a certain value, the stomatal conductance decreased or the stomata closed to avoid xylem embolisms (Franks et al., 2007; Whitehead & Beadle, 2004). In this study, the normalized *F*_d_ for *P. tabulaeformis* and *H. rhamnoides* fitted well with transpiration (VT) when an exponential saturation function was used (Formula (4)). Generally, *F*_d_ increased in response to a rising VT, whereas these values tended to stabilize when VT reached about 50 kPa (W/m^2^)^1/2^ (Figure 8). When VT was <50 kPa, normalized *F*_d_ increased with the increasing of VT, and the canopy conductance also increased, corresponding to the increasing ratio of *F*_d_ and VT. However, when VT was >50 kPa, normalized *F*_d_ tended to stabilize, resulting gradually decreased in the canopy conductance, corresponding to the decreasing ratio of *F*_d_ and VT. Furthermore, the diurnal courses of the estimated relative canopy conductance for *P. tabulaeformis* and *H. rhamnoides* exhibited a single‐peak curve before and after rainfall (Figure 7). These two tree species showed rapidly rising and declining canopy conductance in the morning, but the levels remained low in the afternoon. The results indicated that these two tree species regulate their conductance in response to high VT values (Köcher, Gebauer, Horna, & Leuschner, 2009). In addition, the normalized *F*_d_ for these two tree species slowly increased with VT before rainfall, indicating that the ability of the tree to transmit water was low and transpiration was restrained, but this situation improved slightly after rainfall, which may be explained by a release of xylem hydraulic conductivity under the temporal recharge of soil water (Eberbach & Burrows, 2006; Pataki et al., 2003).

The large difference in each coefficient (parameters *a* or *b*) before and after rainfall in *H. rhamnoides* suggests that there was a high water demand and that soil water conditions had a major influence on the transpiration process (Table 2). In contrast, *P. tabulaeformis* was less sensitive to soil moisture change (Figure 5a). The fine root distribution characteristics may be a reason for the sensitive response to soil water changes (Kume, Takizawa, Yoshifuji, & Suzuki, 2007). A preliminary survey of the fine root distribution in the soil profiles at the study sites showed that *P. tabulaeformis* roots tended to be concentrated at around 10 and 40 cm depth, respectively; *H. rhamnoides* roots were concentrated around 50 cm depth. Furthermore, the surface area of *P. tabulaeformis* fine roots was greater than that of *H. rhamnoides* at the same soil depth (Figure 3). However, the relationship between the presence of roots in a particular soil layer and the magnitude of their contribution to water absorption remains unclear (Moreira, Sternberg, & Nepstad, 2000). Therefore, in the further study, the plant root dynamics need to be investigated, and the hydrogen and oxygen stable isotopes should be used to quantify the water sources along soil profile (West et al., 2012). Meanwhile, the differences in the biological characteristics of conifers and broad‐leaf trees (e.g., the leaf anatomical structures and xylem anatomical characteristics) may also affect the sensitive responses to soil moisture recovery (Sun, Sun, Wang, & Zhou, 2005). Furthermore, the different sensitivity of sap flow responses to temporary recharge may also come from species‐specific effects, such as the stomatal regulation of transpiration (Köcher et al., 2009). Several studies have found a trade‐off relationship between tree water transport, leaf anatomical structure, stomatal conductance, and the distribution of root systems in ecosystems with water shortages, which would enable tree species to reach a homeostasis equilibrium and adapt to the environment under climate change (Sun et al., 2005).

### 4.3 Types of water use

According to McDowell et al. (2008), the terms “isohydric” and “anisohydric” are used to divide the continuum of stomatal regulation of water status into two categories. Isohydry is generally attributed to strong stomatal control of the transpiration rate, which results in the observed similarity in ψ_m_. Isohydric behavior has been observed in temperature hardwoods, C_4_ grasses, Australasian and neotropical trees, and other species of gymnosperms (Bonal & Guehl, 2001; Fisher, Williams, Do Vale, Da Costa, & Meir, 2006). Anisohydry typically exhibits less stomatal sensitivity to soil moisture, allowing large fluctuations in ψ_m_. Anisohydric behavior has also been observed in sugar maple (*Acer saccharum*), sunflower (*Helianthus annuus*), and eucalyptus (*Eucalyptus gomphocephala*) (Franks et al., 2007; West et al., 2008). Our results showed that during the study period, as the soil water decreased (Figure 1e,f), *P. tabulaeformis* reduced *G*_s_ to maintain a relatively constant ψ_m_. In contrast, *H. rhamnoides* allowed large fluctuations in ψ_m_ to maintain *G*_s_ (Figure 9). Therefore, *P. tabulaeformis* should be considered an isohydric species, while *H. rhamnoides* should be considered an anisohydric species.

Anisohydric and isohydric regulation of water status may be a critical factor in the regulation of survival and mortality during drought (McDowell et al., 2008). Compared with isohydric species, anisohydric species tend to occupy more drought‐prone habitats to some extent. In this study, soil water content decreased from June to August (Figure 1e,f). Meanwhile, June to August represents a period of vigorous growth for trees, resulting in great demand for soil water. Therefore, under short‐term drought conditions, *P. tabulaeformis* and *H. rhamnoides* may use a continuum of stomatal regulation of water status to maintain their growth, but under long‐term and intense drought conditions, the advantages of isohydry are readily apparent. The stomatal control system prevents their xylem water potential from falling below a critical threshold, which is an advantage in an environment with fluctuating evaporative demand or soil moisture (Franks et al., 2007).

## 5 CONCLUSIONS

Due to low rainfall and high atmospheric evaporative demands, tree species in arid and semiarid regions experience water shortages during the course of their life cycle. This study discussed the effects of summer rainfall on the main tree species in the Loess hilly region. Results showed differences in sap flow characteristics and responses to rainfall for *P. tabulaeformis* and *H. rhamnoides*. *H. rhamnoides* belongs to an anisohydric type, as *F*_d_ in this species is more sensitive to rainfall and allows large fluctuations in ψ_m_ to maintain *G*_s_ with soil moisture decreases. In contrast, *P. tabulaeformis* can be categorized into an isohydric type, which mainly reduces *G*_s_ to maintain a relatively constant ψ_m_. The results have implications for evaluating water use by different tree species, creating regional hydrological models, and selecting sustainable reforestation species in the Loess Plateau. The water use characteristics in the context of changes in rainfall pattern changes need further investigation to understand the long‐term hydrological regime of these species.

## CONFLICT OF INTEREST

None declared.

## AUTHORS CONTRIBUTION

Xu Wu performed the experiment, analyzed data, and wrote the manuscript. YKT and YMC designed the experiment and edited the manuscript. All authors read and approved the final manuscript.
